# Causes and consequences of sympathoexcitation across the lifespan: Physiological or pathological?

**DOI:** 10.1113/EP091217

**Published:** 2023-09-15

**Authors:** Zoe H. Adams, Jill N. Barnes, Rachel N. Lord

**Affiliations:** ^1^ Cardiff School of Sport and Health Sciences Cardiff Metropolitan University Cardiff UK; ^2^ Bruno Balke Biodynamics Laboratory, Department of Kinesiology University of Wisconsin Madison Madison WI USA

1

The role of the sympathetic nervous system in buffering acute changes in blood pressure is well established. More recently, attention has turned to the importance of sympathetic activity in chronic regulation of blood pressure (Lohmeier & Iliescu, 2015) and the potential risks associated with excess sympathoexcitation. Elevated sympathetic nerve activity is observed in hypertension (Grassi et al., 2018), heart failure (Grassi et al., 2021), obesity (Alvarez et al., 2002) and metabolic syndrome (Quarti Trevano et al., 2020). Indeed, sympathoexcitation is considered (by some) to be causative in hypertension (Esler et al., 2010; Koeners et al., 2016). As such, the mechanisms driving sympathoexcitation in various patient populations are the focus of ongoing research.

Whether excess sympathoexcitation is always pathological is debated. Progressive sympathoexcitation is a feature of ageing (Fagius & Wallin, 1993; Keir et al., 2020). This sympathetic activation has been suggested to drive the rise in blood pressure that occurs with ageing in most of the global population (Mills et al., 2016). However, given that not all adults become hypertensive with age, the link between age‐related sympathoexcitation and blood pressure has not been fully understood. Furthermore, given the influence of sex hormones on sympathetic blood pressure control (Barnes et al., 2014; Hart et al., 2011), they are an additional factor in age‐related sympathoexcitation. The low oestrogen environment after menopause likely contributes to, and enhances the vasoconstrictive effect of, age‐related sympathoexcitation in ageing females. As such, populations with altered hormone profiles, for example polycystic ovary syndrome, may also experience excess sympathoexcitation, which could contribute to the increased cardiovascular risk observed in these cohorts (Berni et al., 2021). Finally, pregnancy is associated with a large increase in resting sympathetic activity that returns to normal after delivery. This sympathoexcitation appears to be physiological in healthy pregnancies but can be excessive in complicated pregnancies. As such, there remains much to be understood about sympathoexcitation in different populations and conditions. This was the focus of the symposium ‘Causes and consequences of sympathoexcitation across the lifespan: Physiological or pathological?’, which took place at Experimental Biology 2022.

Recent work by Rachel Lord and colleagues has provided insight into whether the age‐related rise in sympathetic nerve activity is related to poorer baroreceptor function in later life. Their review (Hughes et al., 2023) discusses how recent advancements in vascular ultrasound methods can be used to quantify parameters of baroreceptor function, such as rate of unloading and time spent unloaded. Using these novel techniques, Lord et al. (2020) found that baroreceptor unloading mechanics were indeed altered in older age, with a reduced rate of unloading and less time spent unloaded observed in older versus younger healthy males. However, unloading mechanics were related to sympathetic baroreceptor sensitivity function (slope and operating point) only in the younger group (Lord et al., 2020). As such, it appears that an alternative mechanism may be driving age‐related sympathoexcitation in males, given that older males have greater resting sympathetic activity than younger males, despite shorter time spent with unloaded baroreceptors. Importantly, the relationship between baroreceptor unloading mechanics and age‐related sympathoexcitation has yet to be assessed in females. Given the reported sex differences in arterial stiffness (Doonan et al., 2013), baroreceptor mechanics may play a different role in baroreflex function in ageing females.

In their symposium review (Brislane et al., 2022), Craig Steinbeck and colleagues discuss the sympathoexcitation that occurs in pregnancy. Uncomplicated pregnancy is associated with profound changes to blood pressure regulation, where substantial increases in blood volume, sympathetic nerve activity and cardiac output are observed alongside a reduction in arterial pressure. This makes pregnancy a rare period of life in which chronic sympathoexcitation appears to be physiological rather than pathological (Brislane et al., 2022). The mechanisms driving sympathoexcitation in pregnancy are still unclear. There is a large vasodilatation observed in pregnancy, thought to be caused by elevations in vasodilators (nitric oxide and relaxin), which may trigger sympathoexcitation via the arterial baroreflex. Alternatively, elevated sympathetic activity could be driven more directly by pregnancy itself. As Brislane et al. discuss, sympathetic activation can be even greater in pregnancies complicated by comorbidities such as obesity, gestational hypertension and obstructive sleep apnoea. Recent data indicate a causal link between sympathoexcitation and gestational hypertension, where excess elevations in sympathetic activity were observed in females with gestational hypertension versus those with uncomplicated pregnancies before any group difference in mean arterial pressure was observed (Badrov et al., 2019). Therefore, understanding physiological versus pathophysiological sympathoexcitation in pregnancy may be key to determining which individuals are at increased risk of complicated pregnancy.

Finally, Charlotte Usselman and colleagues discuss the role of sympathoexcitation in elevated cardiovascular disease risk in polycystic ovary syndrome (PCOS) (Duval et al., 2023). An endocrine condition characterised by excess androgen hormones, PCOS often presents with symptoms of hirsutism and irregular menstruation. It is now established that PCOS patients are also at increased risk of hypertension, cardiovascular events, obesity and insulin resistance compared to healthy controls (Berni et al., 2021), although the mechanisms underlying this increased risk remain poorly understood (Duval et al., 2023). To date, there is mixed evidence on whether sympathetic nerve activity is chronically elevated in PCOS, although several studies have reported chronic sympathoexcitation in this population. Additionally, endothelial function appears to be poorer, and arterial stiffness greater, in PCOS versus healthy volunteers. Whether these differences are driven by altered sympathetic control of the vasculature, and the contribution of hyperandrogenism to sympathetic regulation, remains unclear. Interestingly, based on evidence in animal models of PCOS, Duval et al. discuss the possibility that sympathoexcitation may actually *drive* PCOS development and severity. Inhibition of sympathetic activity directed to the ovaries reduced symptoms in a rat model of PCOS (Del Campo et al., 2020), suggesting a possible role of sympathoexcitation in PCOS development. Although of limited applicability to human PCOS, these data support an interesting interpretation of the sympathoexcitation observed in PCOS. Determining the mechanisms that drive sympathetic activation in PCOS patients could suggest potential targets for reducing cardiovascular risk in this population.

As these varied symposium reviews show, chronic sympathoexcitation is not simply a risk factor for cardiovascular disease. The mechanisms driving sympathetic activation are population and disease specific, and determining the cause and impact of pathological sympathoexcitation in various populations is the focus of ongoing research. Crucially, all three reviews highlight the lack of understanding surrounding the role of chronic sympathoexcitation in female cardiovascular health. They demonstrate the need for physiological research to be conducted in the context of sex hormones and sex‐specific conditions in order to make meaningful improvements to female health.

## AUTHOR CONTRIBUTIONS

Rachel N. Lord conveived of the work. All authors drafted the work or revised it critically for important intellectual content. All authors approve the final version of the manuscript and agree to be accontable for all aspects of the work in ensuring that questions related to the accuracy or integrity of any part of the work are appropriately investigated and resolved. All persons designated as authors qualify for authorship, and all those who qualify for authorship are listed.

## CONFLICT OF INTEREST

The authors declare no conflicts of interest.

## FUNDING INFORMATION

No funding was received for this work.
